# Mesoporous Silica Materials Loaded with Gallic Acid with Antimicrobial Potential

**DOI:** 10.3390/nano12101648

**Published:** 2022-05-12

**Authors:** Gabriela Petrisor, Denisa Ficai, Ludmila Motelica, Roxana Doina Trusca, Alexandra Cătălina Bîrcă, Bogdan Stefan Vasile, Georgeta Voicu, Ovidiu Cristian Oprea, Augustin Semenescu, Anton Ficai, Mircea Ionut Popitiu, Irina Fierascu, Radu Claudiu Fierascu, Elena Lacramioara Radu, Lilia Matei, Laura Denisa Dragu, Ioana Madalina Pitica, Mihaela Economescu, Coralia Bleotu

**Affiliations:** 1 Science and Engineering of Oxide Materials and Nanomaterials, Faculty of Applied Chemistry and Materials Science, University POLITEHNICA of Bucharest, Gh. Polizu 1-7, 011061 Bucharest, Romania; gabriela.petrisor06@yahoo.com (G.P.); motelica_ludmila@yahoo.com (L.M.); truscaroxana@yahoo.com (R.D.T.); ada_birca@yahoo.com (A.C.B.); bogdan.vasile@upb.ro (B.S.V.); georgeta.voicu@upb.ro (G.V.); anton.ficai@upb.ro (A.F.); 2 National Research Center for Food Safety, University POLITEHNICA of Bucharest, Splaiul Independentei 313, 060042 Bucharest, Romania; ovidiu73@yahoo.com; 3 National Center for Micro and Nanomaterials, University POLITEHNICA of Bucharest, Splaiul Independentei 313, 060042 Bucharest, Romania; 4 Department of Inorganic Chemistry, Physical Chemistry and Electrochemistry, Faculty of Applied Chemistry and Materials Science, University POLITEHNICA of Bucharest, Gh. Polizu 1-7, 011061 Bucharest, Romania; 5 Department Engineering and Management for Transports, University POLITEHNICA of Bucharest, 060042 Bucharest, Romania; augustin.semenescu@upb.ro; 6 Academy of Romanian Scientists, Ilfov Street 3, 050044 Bucharest, Romania; 7 Department of Vascular Surgery and Reconstructive Microsurgery, Victor Babes University of Medicine and Pharmacy, Timisoara, Piata Eftimie Murgu, Nr. 2, 300041 Timisoara, Romania; mirceapopitiu@yahoo.com; 8 National Institute for Research & Development in Chemistry and Petrochemistry—ICECHIM, Splaiul Independentei 202, 060021 Bucharest, Romania; dumitriu.irina@yahoo.com (I.F.); radu_claudiu_fierascu@yahoo.com (R.C.F.); 9 University of Agronomic Sciences and Veterinary Medicine of Bucharest, 011464 Bucharest, Romania; 10 Stefan S. Nicolau Institute of Virology, Mihai Bravu 285, 030304 Bucharest, Romania; lacramioara.radu@virology.ro (E.L.R.); lilia.matei@virology.ro (L.M.); denisa.dragu@virology.ro (L.D.D.); ioana.madalina.pitica@virology.ro (I.M.P.); mihaela.economescu@virology.ro (M.E.); cbleotu@yahoo.com (C.B.)

**Keywords:** mesoporous materials, soft template, gallic acid, drug delivery, antimicrobial, dysbiosis, inflammasome

## Abstract

This paper aimed to develop two types of support materials with a mesoporous structure of mobile crystalline matter (known in the literature as MCM, namely MCM-41 and MCM-48) and to load them with gallic acid. Soft templating methodology was chosen for the preparation of the mesoporous structures—the cylindrical micelles with certain structural characteristics being formed due to the hydrophilic and hydrophobic intermolecular forces which occur between the molecules of the surfactants (cetyltrimethylammonium bromide—CTAB) when a minimal micellar ionic concentration is reached. These mesoporous supports were loaded with gallic acid using three different types of MCM—gallic acid ratios (1:0.41; 1:0.82 and 1:1.21)—and their characterizations by FTIR, SEM, XRD, BET and drug release were performed. It is worth mentioning that the loading was carried out using a vacuum-assisted methodology: the mesoporous materials are firstly kept under vacuum at ~0.1 barr for 30 min followed by the addition of the polyphenol solutions. The concentration of the solutions was adapted such that the final volume covered the wet mesoporous support and—in this case—upon reaching normal atmospheric pressure, the solution was pushed inside the pores, and thus the polyphenols were mainly loaded inside the pores. Based on the S_BET_ data, it can be seen that the specific surface area decreased considerably with the increasing ratio of gallic acid; the specific surface area decreased 3.07 and 4.25 times for MCM-41 and MCM-48, respectively. The sample with the highest polyphenol content was further evaluated from a biological point of view, alone or in association with amoxicillin administration. As expected, the MCM-41 and MCM-48 were not protective against infections—but, due to the loading of the gallic acid, a potentiated inhibition was recorded for the tested gram-negative bacterial strains. Moreover, it is important to mention that these systems can be efficient solutions for the recovery of the gut microbiota after exposure to antibiotics, for instance.

## 1. Introduction

Drug delivery systems have gained a great deal of interest for medical applications, being suitable for the treatment of cancer, tissue engineering, bone regeneration, infections or osteoporosis, treatment of neurodegenerative or inflammatory diseases [1,2,3,4], etc., as they can ensure smart delivery at the desired site and may also optimize the release of the desired biologically active agent and reduce or eliminate systemic toxicity. Triggers are pH, temperature, presence of enzymes, magnetic fields, light, etc. [5,6,7,8,9,10].

In recent years, nanotechnology has become a promising approach for developing nanostructured materials as new systems for transporting and releasing biologically active agents such as drugs, cytostatics, proteins, natural substances, etc. Mesoporous silica nanoparticles (MSNs) have special structural characteristics, such as an ordered porous structure (with control of the loading and release kinetics of the drug), a large specific surface area and pore volume that can be selectively functionalized (over 1000 m^2^/g and over 1 cm^3^/g), good biocompatibility, and high chemical and thermal stability—making MSNs an optimal material for their use as transport and administration systems for drugs, and thus being suitable for addressing challenging biomedical applications in both therapy and imaging [1,11,12,13,14,15,16,17,18,19].

The literature describes many synthesis techniques for MSNs, such as the sol-gel method, hydrothermal synthesis, microwave synthesis, and the template method (using soft or hard templates) [20]. Currently, the synthesis of MSNs has undergone various changes and is mainly based on the judicious choice of the nature and concentration of the surfactant (template) or solution parameters (temperature, pH, presence of co-surfactant, etc.), resulting in mesoporous materials with different morphologies, sizes, and also pore characteristics—especially pore size, orientation, etc. For MSNs to be ideal carriers for drug delivery, the pore size must be uniform, the ratio of molecule size to pore size should be within a certain range, and the pore volume should be large to increase the loading capacity [19,21,22].

Their unique physicochemical properties have made these mesoporous materials excellent candidates for drug delivery applications. MCM-41 and MCM-48 are part of the MSNs family and can encapsulate large molecules in their structural pores and delivery them to a target with greater accuracy [22].

Mesoporous materials, obtained through various synthesis techniques, provide a very important tool for the controlled release of various biologically active compounds involved in various metabolic processes. Among the possible biologically active compounds, polyphenols (secondary metabolites of plants, comprising over 8000 types of structures) stand out from synthetic compounds, having the advantage of using natural resources within the concept of “green chemistry”. From this category of polyphenols, gallic acid (one of the most widespread phenolic acids found in both fruits and herbs) has a high potential for various applications due to its antioxidant, antimicrobial, antitumor, anti-inflammatory, antimelanogenic, anti-allergic, neuroprotective, and hepatoprotective effects. Polyphenols, in general, can be considered efficient prebiotics and are increasingly used for their benefits [23,24,25,26,27,28,29,30,31]. As is the case with the vast majority of natural products, the use of the polyphenols mentioned above is limited due to their metabolism and rapid elimination, resulting in low bioavailability. Thus, it the need to develop transport systems that simultaneously ensure both the protection and controlled release of these polyphenols is obvious [31,32]. 

In this work, we loaded gallic acid via a vacuum-assisted methodology into the pores of the two mesoporous materials, MCM-41 and MCM-48, and then their release and biological activity were assessed. The gallic acid-based mesoporous systems were efficient in reducing the inflammatory response and could be used in microbiota recovery.

## 2. Experimental Methods

### 2.1. Chemicals and Apparatus

**Figure 1:**
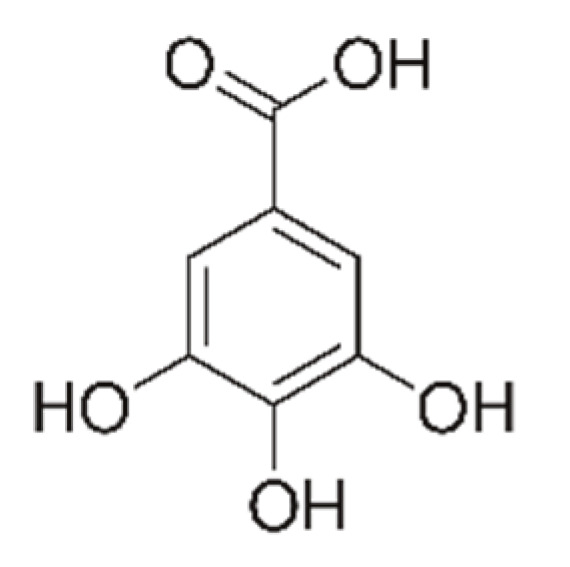
Structure of gallic acid.

For the synthesis of systems, gallic acid (Figure 1, Merck, Darmstadt, Germany), N-cetyl-N,N,N-trimethylammonium bromide (CTAB, Merck), tetraethyl orthosilicate (TEOS, Sigma-Aldrich Co. Merck Group, Darmstadt, Germany), ammonia (S.C. Silal Trading SRL, Bucharest, Romania), acetone (Sigma-Aldrich Co. Merck Group) and ethanol (Sigma-Aldrich Co. Merck Group) were used reagents grade and without further purification. Distilled water was used throughout the experiments.

For the release study, Sodium chloride (NaCl, Sigma-Aldrich Co. Merck Group) and acetonitrile HPLC grade (Sigma-Aldrich Co. Merck Group), trifluoroacetic acid HPLC grade (TFA, Sigma-Aldrich Co. Merck Group), sodium hydroxide 1N (NaOH, S.C. Silal Trading SRL) and hydrochloric acid 2N (HCl, S.C. Silal Trading SRL), and potassium hydrogen phosphate 99% (KH_2_PO_4_, Carl Roth, Karlsruhe, Germany) were used.

The MCM-41-based mesoporous systems were characterized using specific physico-chemical methods: X-ray diffraction (XRD), nitrogen adsorption and desorption studies—BET (Brunauer–Emmett–Teller), Fourier transform infrared spectroscopy (FTIR), scanning electron microscopy (SEM), and transmission electron microscopy (TEM). XRD spectra were recorded on Panalytical X’Pert Pro MPD equipment, with Cu-Kα radiation. FTIR analysis was performed using a Thermo IN50 MX. BET analysis was performed on a Micrometrics Gemini V surface area and pore size analyzer. Magnetization curves were recorded using a Vibrating Sample Magnetometer VSM-7304 Lake Shore (USA) operated up to 12,000 Oe. FTIR microscope operated in reflection mode was carried out to study structural features. The surface morphology of the samples was examined via a QUANTA INSPECT F electron microscope equipped with a field emission gun and an energy dispersive (EDS) detector on samples covered with silver. The transmission electron microscopy images were obtained on finely powdered samples using a Tecnai™ G2 F30 S-TWIN high-resolution transmission electron microscope (HRTEM) from FEI equipped with selected area electron diffraction (SAED). The microscope was operated in transmission mode at 300 kV with a TEM point resolution of 2 Å and line resolution of 1 Å. Thermogravimetric analyzes were recorded using a Netzsch 449C STA Jupiter instrument from room temperature up to 900 °C, in an open alumina crucible, at a heating rate of 10 °C/min in dry air (50 mL/min).

### 2.2. Preparation of the Mesoporous Materials

Two mesoporous materials were obtained using the soft templating route. The synthesis of the two mesoporous silica materials used the same surfactant—namely cetyltrimethylammonium bromide (CTAB)—which, under the basic conditions, aims to assemble into cylindrical micelles, which are further organized into a hexagonal or cubic structure. Around these structures, the silica network is formed by classical hydrolysis and condensation reactions. The template removal by calcination represents the final step. 

The mesoporous materials were obtained as follows: 0.5 g CTAB was dissolved in 96 mL distilled water and sonicated until the solution became clear. Subsequently, 34 mL of ethanol and 10 mL of an ammonia solution were added and stirring was continued until the solution became homogeneous. After mixing, 2 mL of TEOS was added and stirred for an additional three hours at the same speed of rotation and a translucent precipitate was obtained. The precipitate thus obtained was filtered and washed with distilled water and ethanol. The last step of the synthesis consisted of purification/washing with ethanol (20 mL) and water (three times with 20 mL) and drying for 12 h at 100 °C. The final product was annealed for nine hours at 550 °C according to the following program: 0–300 °C at 80 °C /min and 300–550 °C at 20 °C /min. This mesoporous material was labelled MCM-41. Similarly, but using 2 g of CTAB, another mesoporous silica material was obtained and labelled MCM-48.

### 2.3. Adsorption of Gallic Acid on Mesoporous Materials

The adsorption of gallic acid was carried out by contacting the mesoporous materials to the gallic acid solution in acetone (saturated solution). In order to assure a good loading of gallic acid inside the porous system, the loading was carried out under vacuum at room temperature. Prior to adding the gallic acid solution, the MCM-41 was exposed for 30 min at a 100 mbar vacuum. During this period, the air and water traces were partially removed from the pore system, and when the solution was added and the bottle pressurized, the solution was practically absorbed into the pores of the mesoporous material. The obtained samples were dried at 100 °C. The amount of gallic acid loaded from the systems obtained (Table 1) was evaluated by complex thermal analysis.

### 2.4. Release Studies

The release of gallic acid from the mesoporous silica was studied in two simulated biological fluids [31]: simulated gastric fluid (SGF) with pH = 1.2 and simulated intestinal fluid (SIF) with pH = 6.8, considering the intention to use the materials for oral administration or in the form of suppositories. Release studies were performed at a constant temperature of 37 ± 1 °C and a magnetic stirring of 270 rpm. Working conditions are presented in Table 2.

The amount of active substance released was determined using liquid chromatography using an Agilent liquid chromatograph with DAD-type UV-Vis detector. In order to determine the amount of active substance in the samples, calibration curves were made for gallic acid in the two simulated biological fluids (SGF and SIF) in areas of adequate concentrations relative to the theoretical concentrations of gallic acid.

### 2.5. Cell Viability

The CellTiter-Glo viability test was performed according to the manufacturer’s instructions (Promega, catalogue number: G7570). Briefly, the HT29 cells (ATCC^®^ HTB-38) were seeded in triplicate, at a 7500 cells/well concentration using 96-well opaque plates. After incubation for 24 h, the cells were treated with gallic acid and its combinations with MCM-41 or MCM-48 in concentrations ranging from 1000 µg/mL to 7.82 µg/mL for 24 h. The cells were then incubated for 10 min with CellTiter-Glo reagent, and the luminescence was measured using a 96-well microplate multi-reader Tristar2S LB942 (Berthold Technologies, Bad Wildbad, Germany). Background luminescence was measured in a cell-free medium and subtracted from experimental values.

### 2.6. Caspase-1 Activation Test

The Caspase-Glo^®^ 1 Inflammasome Assay (Promega, Madison, WI, USA) was used to detect activated caspase-1 in HT29 cells (ATCC^®^ HTB-38). The cells were seeded in 96-well microplates with a clear, opaque white bottom and were maintained at 37 °C for 24 h. HT29 cells were seeded at a concentration of 7500 cells/well. The prebiotics were diluted in Tryptic Soy Broth medium to a 2 mg/mL concentration, and E. coli was subsequently added at a concentration of 1 McFarland standard. After 4 h, 10 µL of prebiotic-treated bacteria was added to the cells. In other columns, 10 μL of prebiotics diluted in the medium at a 2 mg/mL concentration were added. The Caspase-Glo^®^ 1 reagent was prepared and added to the wells of the 96-well plate according to the manufacturer’s instructions. The plates were vortexed for 30 s at 500 rpm and incubated for 2.5 h to stabilize the luminescent signal. The luminescence was read at half-hour intervals (to determine when to stop the reaction—Maximum luminescence) with the Tristar2S LB942 microplate multi-reader (Berthold Technologies). 

### 2.7. RNA Expression

HT29 cells were seeded in 6-well plates at a concentration of 3.5 × 10^5^ cells/well. At 24 h, the cells were treated with 200 µg/mL of GA combinations with mesoporous silica. The next day, the *E. coli* bacterium was added at a concentration of 0.5 McFarland standard and kept in the cellular microenvironment for 4 h (at which time the bacterial infection could be observed under an optical microscope). Total RNA was extracted from cells with Trizol reagent followed by chloroform extraction and isopropanol precipitation. A total of 2 μg of the resulting RNA was reverse-transcribed into cDNA using random hexamers and MML-V reverse transcriptase in a total volume of 20 μL, according to the manufacturer’s protocol. Quantitative PCR was performed with Maxima SybrGreen Supermix and primers for NLRP3, IL-1, Casp1, GASD, RIPK1, IL18, Casp3, Casp7, Casp8, Casp9, cIAP1, cIAP2, PON2, IL8, IFNg, LAMA4, CLDN3, OCL TJP1, and CSTB. The induction of expression above control levels was calculated by the delta-delta-Ct method.

### 2.8. Evaluation of the Effects of Prebiotics against Bacterial Strains (MICs)

The effects of prebiotics against bacterial strains were tested by the broth micro-dilution method using 96-well plates and Muller Hinton broth (MHB), according to the Clinical and Laboratory Standards Institute (CLSI) guidelines for MIC breakpoints [33]. The following bacterial strains were used for the prebiotic-effect evaluations: *E. coli* 25922, *E. coli* ESBL GG3048, *Pseudomonas aeruginosa, Enterococcus faecium, Salmonella tiphymurium,* and *Shigella flexneri*. The prebiotics was diluted in the medium to between 1 mg/mL and 7.8 µg/mL. Over 90 µL of prebiotic dilution and 10 µL of the 24 h bacterial culture, with a cell density of 0.5 McFarland standard, was added. The plates were incubated at 37 °C for 24 h. After incubation, the plates were observed for visible growth and evaluated spectrophotometrically at 620 nm. The MIC was interpreted as the lowest antibiotic/prebiotic concentration at which no visible increase was observed.

### 2.9. Synergy Experiments with Prebiotics with AMX (Fractional Inhibitory Concentration)

The interactions of the antimicrobial agents with gallic acid were investigated by the chessboard method using 96-well microtiter plates, according to the slightly modified method of Vipin C. et al. [34]. Various bacterial strains were inoculated into separate 96-well plates. The stock of amoxicillin (AMX; 10 mg/mL) and the stock of gallic acid (10 mg/mL) were added to the wells to obtain the required antibiotic and prebiotic concentrations. Thus, each well of the 96-well plate contained 10 μL of a bacterial suspension at a concentration of 0.5 McFarland standard, 10 μL of prebiotic suspension with a concentration of 150 μg/mL or 500 μg/mL, and fractional doses of AMX (binary dilutions), so as to obtain a final antibiotic concentration of between 1 mg/mL and 0.97 µg/mL. The contents were incubated at 37 °C for 24 h and observed for visible turbidity. A control consisting of 0.5 McFarland standard antimicrobial agents were incubated separately for comparison. The experiment also included a control for prebiotic effects (treatment of bacteria with prebiotics) and an untreated bacteria control. The CMI of the AMX in combination with prebiotics was defined as the lowest concentration at which no visible growth was observed after 24 h of incubation. Bacterial concentration was determined spectrophotometrically, and the decrease in the current absorbance of the prebiotic compared to the inhibition of bacterial growth in the presence of AMX only was considered to be the prebiotic intake.

## 3. Results and Discussion

All the materials were characterized by characteristic physico-chemical methods:

### 3.1. XRD Diffraction

**Figure 2:**
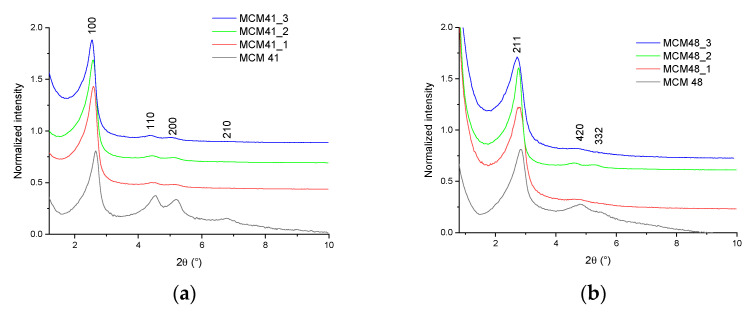
XRD diffractograms of samples: (**a**) MCM-41, MCM-41_1, MCM-41_2, MCM-41_3; (**b**) MCM-48, MCM-48_1, MCM-48_2, MCM-48_3.

Sample diffractograms were interpreted based on comparisons with data from the literature [35,36,37,38]. Additionally, in order to be able to study the variation of some characteristics of the diffractograms, they were normalized at the height of the most intense peak present (Figure 2a,b).

All diffractograms recorded on MCM-41 mesoporous materials showed four diffraction peaks corresponding to the crystallization planes (100), (110), (200), and (210), characteristic of the ordered hexagonal structure characteristic of MCM41 materials—one of strong (100) and three of low intensity (110), (200), and (210). These peaks were consistent with the literature data [35]. The decrease in the intensity of the peaks characteristic of crystallization planes 110, 200, and 210 from the diffractograms of mesoporous materials of MCM-41 type loaded with gallic acid may indicate a slight decrease in pore diameter, as a result of the introduction of polyphenols into MCM-41 pores—but may also indicate a possible deposition (in smaller quantities) on the surface of the material. This behavior was visible when evaluating the diffractograms normalized to the value of the peak intensity corresponding to the plane 110 (Figure 2), observing the disappearance of the peak corresponding to plane 210 accompanied by the sharp decrease of the intensity of the other peaks. There was also a slight displacement of the peaks in the diffractograms of the MCM-41/polyphenol mesoporous materials compared to the diffraction peak characteristic of the MCM-41 material [36,37]. This behavior can be correlated with a decrease in pore size.

The diffractograms recorded on the MCM-48 mesoporous materials showed two major peaks corresponding to crystallization planes (211) and (420), and one minor peak corresponding to the (332) plane. These peaks are consistent with the literature data [38]. The peaks characteristic of the (211) and (420) crystallization planes from the diffractograms of the mesoporous materials of the MCM-48 type loaded with polyphenols decreased in intensity as a result of their absorption—a phenomenon also very visible for the peak corresponding to the (332) plane. The decrease in intensity and the displacement of the characteristic peaks from the diffractograms of mesoporous materials of the MCM-48 type loaded with polyphenols may indicate a slight decrease in pore diameter, as a result of the introduction of polyphenols into the MCM-48 pores—but may also indicate a possible deposition (in smaller quantities) on the surface of the material. This behavior is visible when evaluating the diffractograms normalized to the value of the peak intensity corresponding to plane (211), observing the disappearance of the peak corresponding to plane (332) accompanied by the sharp decrease of the peak corresponding to the (420) plane (Figure 2b). 

X-ray diffraction cannot directly measure the internal pore size without an independent measure of the wall thickness. However, according to the literature data [39], starting from the assumption of a perfect hexagonal structure, the hexagonal unit cell parameter a0 for MCM-41 (representing the spacing between the hexagonal layers) can be calculated as:

$$a_{0}=d_{100}\left(\frac{2}{\sqrt{3}}\right)$$

representing the average distance between pores, and in the ideal structure presumed, the internal pore diameter plus the thickness of the pore walls. 

The unit cell parameter for MCM-48 can be calculated, according to the literature data [40] as:

$$a_{0}=d_{211}\left(\sqrt{6}\right)$$

For the unloaded samples, the values of the d spacing were 37.51 Å (d100—MCM-41) and 34.52 Å (d211—MCM-48), respectively, resulting in unit cell parameters calculated as 43.31 Å (MCM-41) and 86.54 Å (MCM-48), respectively—in good agreement with the literature data [39,41]

### 3.2. Brunauer–Emmett–Teller (BET)

**Figure 3:**
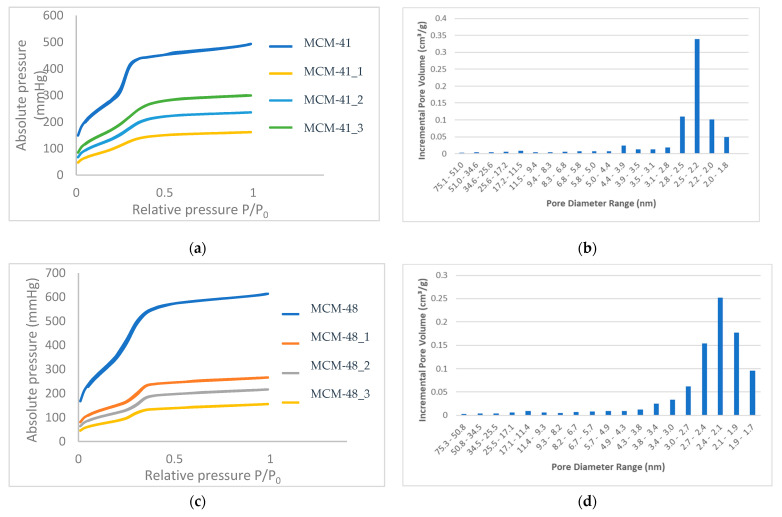
Absorption isotherms recorded on materials (**a**) MCM-41, MCM-41_1-3, and (**c**) MCM-48 and MCM-48_1-3 and pore size distribution histograms for (**b**) MCM-41 and (**d**) MCM-48.

Analyses were performed on 77K Micrometrics Gemini V equipment in liquid nitrogen—the absorption/desorption curves for the samples being shown in Figure 3 and the main pore characteristics (specific surface area and volume of pores) obtained for the mesoporous samples presented in Table 3.

All MCM-41 samples show type IV isotherms with a type H1 hysteresis loop, typical for MCM-41 materials with an ordered hexagonal structure with cylindrical pores of similar size [9]. The high specific surface area of MCM-41 of 1179 m^2^/g decreased with the loading of polyphenols, which means that the polyphenols were largely absorbed into the pores, and perhaps only a small amount was deposited on the surface. A similar behavior was observed for the MCM-48 mesoporous support, as presented in Table 3. As a consequence of the loading, the BET surface area decreased by 3.07 and 4.25 times for the MCM-41 and MCM-48, respectively. 

The nitrogen absorption/desorption isotherms (Figure 3a) of the mesoporous materials MCM-41 and MCM-41_1–3—in the range of relative pressures (P/P_0_) 0.01–0.99—were completely reversible, indicating the uniformity of the size of the unidirectional tubular mesopores characteristic of some ordered mesoporous structures [38], which leads us to the conclusion that the absorption of polyphenols does not change the ordered structure of the pores, but that these pores are loaded with gallic acid, to different extents.

The nitrogen absorption/desorption isotherms (Figure 3c) of the mesoporous materials MCM-48 and MCM-48_1–3 in the range of relative pressures (P/P_0_) 0.01–0.99 showed type IV isotherms and a stage of condensation in the relative pressure range of 0.2 to 0.3, which is correlated with capillary condensation in the pores of mesoporous materials. For both support materials, the pore size distribution was narrow. 

The pore size distribution (Figure 3b,d) of the two mesoporous samples was in the range of 1.8 to 2.8 for MCM-41 (72% of the pore volume was given by these pores) and a small amount of boarded distribution of between 1.7 and 3.8 for the MCM-48 (about 88% of the pore volume was given by these pores).

In conclusion, if we corroborate the two analyses of XRD and BET, we can say that polyphenols are most likely and to a large extent absorbed inside the pores, and that only a small amount could be found on the surface of the material. 

For both mesoporous materials, the volume of the pores was reduced by 73.29 and 74.66%, respectively, which means that these values represent the loading of MCM-41 and MCM-48.

### 3.3. Fourier Transform Infrared Spectroscopy (FTIR)

**Figure 4:**
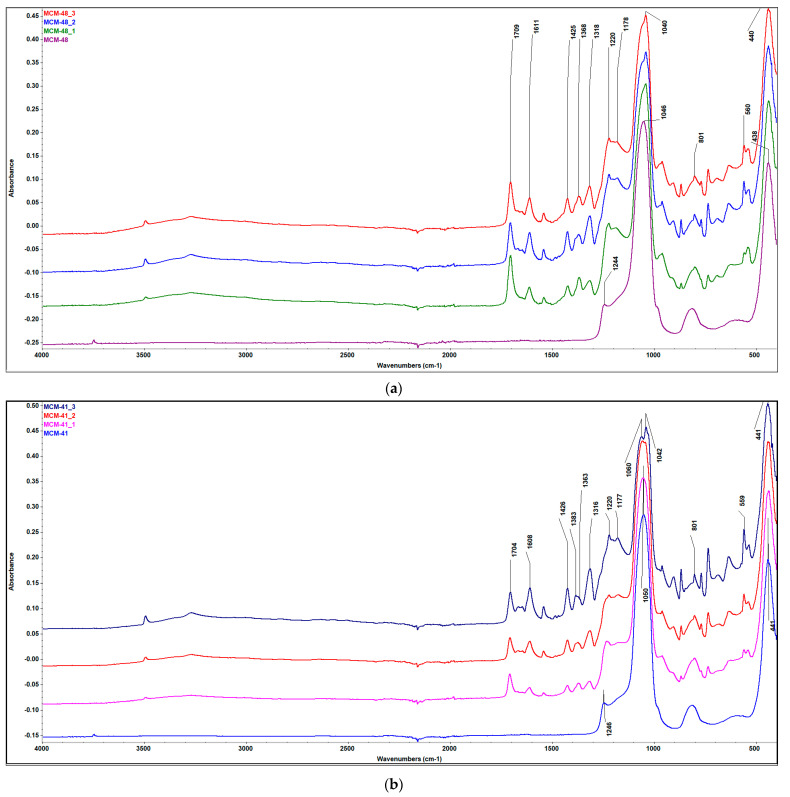
FTIR spectra of samples (**a**) MCM-41, MCM-41_1–3, and (**b**) MCM-48 and MCM-48_1–3.

In the FTIR spectra (Figure 4a) obtained for the mesoporous material of the MCM-41 type, the main characteristic bands of silica can be observed. The bands at ~1245 and 1050 cm^−1^ are characteristic of the tensile vibrations of the asymmetric O–Si–O units, the band at ~443 cm^−1^ is characteristic of the deformation vibrations of the O–Si–O units, the band at ~800 cm^−1^ is characteristic of the tensile vibrations of the symmetrical Si–O–Si units, and the band at ~474 cm^−1^ is characteristic of the deformation vibrations of the symmetrical O–Si–O units. The band from ~960–970 cm^−1^ is associated with the silanolic groups of MCM-41—an extremely important band if the functionalization of mesoporous silica is desired.

In the FTIR spectra obtained for the mesoporous material of MCM-48 (Figure 4b) type, the main characteristic bands of the silica network can be observed. The bands at ~1049 and 1242 cm^−1^ are characteristic of the tensile vibrations of the asymmetric O–Si–O units, the band at ~433 cm^−1^ is characteristic of the deformation vibrations of the O–Si–O units, the band at ~812 cm^−1^ is the characteristic of the tensile vibrations of the symmetrical Si–O–Si units, and the band at ~512 cm^−1^ is characteristic of the deformation vibrations of the symmetrical O–Si–O units. The ~980 cm^−1^ band is associated with the silanol groups of MCM-48.

In the case of the IR spectra for the MCM-41/gallic acid or MCM-48/gallic acid materials (Figure 4a,b), the main bands characteristic of the silica network can be observed, but also the bands characteristic of the organic functional groups in the structure of the absorbed gallic acid. From the FTIR spectrum, it can be seen that when loading the mesoporous material MCM-41 with gallic acid, a series of new bands appear—demonstrating the efficiency of loading with the bioactive compound/polyphenol. Thus, the bands in the area of 3280 cm^–1^ (MCM-41) characteristic of the stretching vibrations of the O–H bonds correspond to the phenolic groups in the polyphenol structure. Additionally, the bands in the range of 1704 and 1615 cm^−1^ correspond to the tensile vibrations of OH and C=O. The bands centered at ~1600 cm^−1^ and ~1366 cm^−1^ result from the tensile vibration of the bond between the benzene nucleus and the carboxyl group in the polyphenol molecule. The band in the ~1426 cm^−1^ area indicates an interaction of the –OH and –COOH groups with the surface of the solid. In addition, CH vibrations of methylene groups in benzene rings of gallic acid can be found in the range of 3000 and 2800 cm^−1^. Additionally, by comparing the FTIR spectra of the mesoporous material synthesized and modified with gallic acid, it is observed that the wide bands in the region of 3280 cm^−1^ (MCM-41), corresponding to the associated OH groups in polyphenols (tensile vibration O–H), became more and more pronounced due to the increase in the concentration of the organic compound (Figure 4a).

By examining the synthesis models for the MCM-41 and MCM-48 samples, it was possible to confirm some internal vibrations of the Si–O bonds and oligomers formed in the synthesis process. For mesoporous structures, it is known that IR bands between 1218 and 1055 cm^−1^ can provide a good indication of the presence of oligomeric species of Si, Si (OSi)^3^, and Si (OSi)^4^ (Q3 and Q4). Based on the literature data [42,43], the strong complex bands observed at ~1218 and 1055 cm^−1^ can be attributed to the internal vibrations of Si–O related to Q3 and Q4 [44]. The 1218 cm^−1^ band is a possible proof of the vibrations of the Q4 external links. The absorption band at ~431 cm^−1^ is attributed to the deformation of Si–O–Si bonds and is also characteristic of mesoporous silica [45,46]. These results are consistent with the vibrational modes of mesoporous silica and related materials [45,46,47,48,49,50,51].

FTIR can also be corroborated with other methods, and some conclusions can be drawn related to the loading of the two supports. First of all, using an ATR technique, the spectra appeared to be a consequence of the surface composition in particular, and thus, the GA loaded inside the pores did not influence the spectra. Even if the ratio between the mesoporous support and the gallic acid was 1:0.4; 1:0.8, and 1:1.2 (the content of the GA increased three times), the relative intensities of the peaks of GA were quite the same; thus, we can confirm that the loading inside the pores was substantial for both the supports and all three concentrations. 

### 3.4. Scanning Electron Microscopy (SEM)

**Figure 5:**
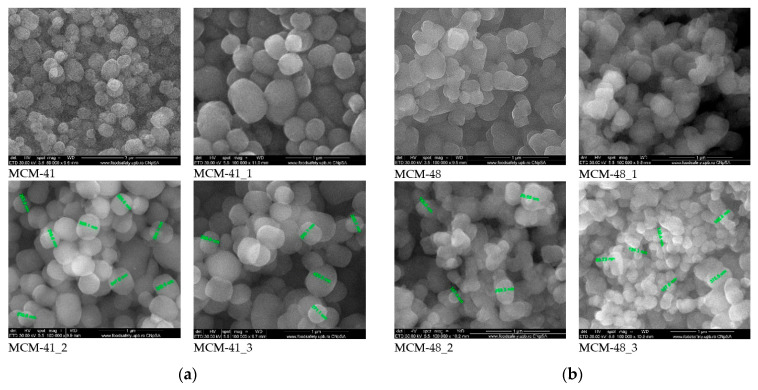
SEM images of (**a**) MCM-41 mesoporous materials loaded with gallic acid and (**b**) MCM-48 mesoporous materials loaded with gallic acid.

All samples were coated with a silver film before analysis. Scanning electron microscopy images (Figure 5a,b) recorded on the mesoporous materials showed the spherical morphology of the silica particles, with variable sizes in the range of 200–400 nm—the size not being influenced by the loading with gallic acid even if the loading capacity exceeded 120%. Compared to the support materials, the SEM images of the samples loaded with gallic acid showed some heterogeneous areas in the form of agglomerates, which can be attributed to the presence of polyphenols on the surface. These agglomerates were much smaller than the spherical particles of the MCM-41 and MCM-48, and their content was low—which means that only a small amount of the gallic acid was deposited onto the surface, this being mostly arranged inside the pores. At high magnification (100,000×), it could be clearly observed that the SEM images of the MCM-41 and 48 samples were more translucent compared to the samples loaded with gallic acid. These observations are in good agreement with the BET analyses. 

### 3.5. Transmission Electron Microscopy (TEM)

**Figure 6:**
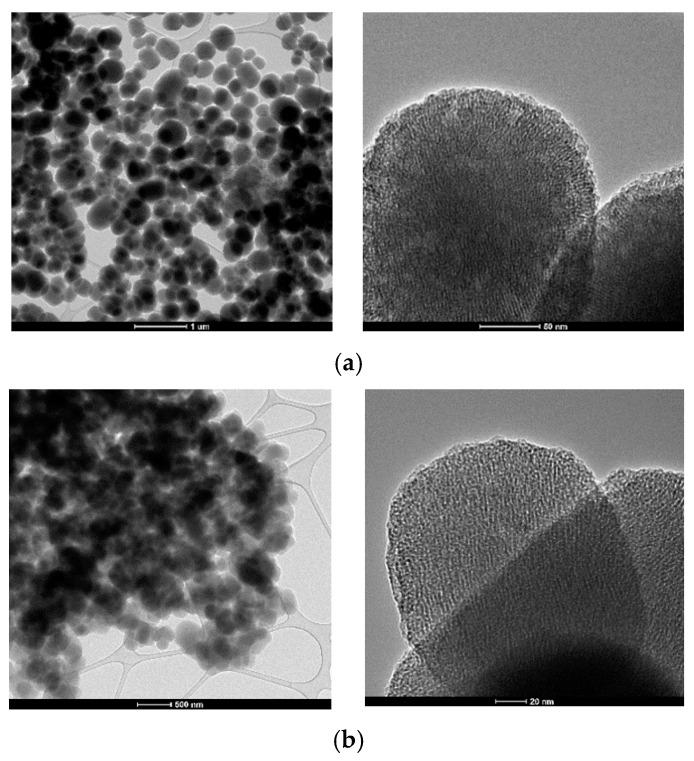
TEM images of the mesoporous supports (**a**) MCM-41 and (**b**) MCM-48 at lower and higher magnification.

Transmission electron microscopy images recorded on the two mesoporous samples, namely MCM-41 and MCM-48, are presented in Figure 6. Based on these images of both the powders at two magnifications, it can be concluded that both samples are spherical, and that the size distribution of these particles is mostly within 200–600 nm (slightly narrower in the case of MCM-48). At the higher magnification, the porous nature can be observed, but the measurement of the size of the pores is difficult because of the non-crystalline nature of the silica materials.

### 3.6. Thermogravimetric Analyses

**Figure 7:**
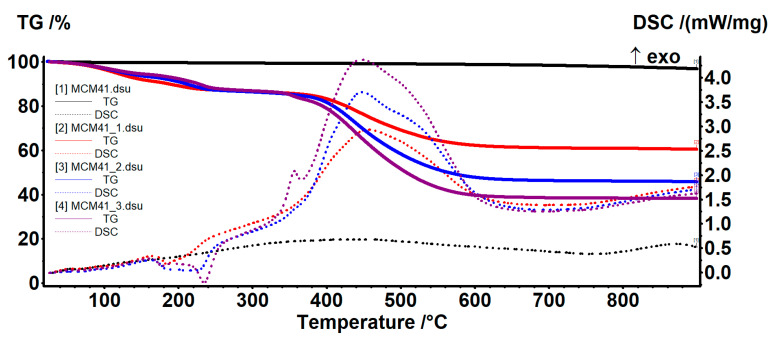
TG-DSC curves for the MCM41 and MCM41-GA samples.

The amount of water absorbed and adsorbed as well as the density of silanol groups on the surface of the nanoparticles were evaluated using thermal analysis. For MCM-41 (Figure 7), there are two temperature ranges assigned in the literature to these two processes (water removal and condensation of silanol groups). Thus, at up to 200 °C, the sample loses the water molecules physically absorbed on its surface and in its pores, as well as the water molecules that physically interact with the surface of the nanoparticles (mainly via hydrogen bonds).

The mass loss recorded in the range of 20–200 °C was 0.57%. At up to ~100 °C, the sample will mainly lose water that is not bound to nanoparticles or has very weak interactions—0.45%. In the range of 100–200 °C, the water molecules that interact with the surface of the MCM41 are eliminated, and therefore require a higher energy to be released—0.12%. The release of these two types of water molecules was highlighted by the endothermic effect on the DSC curve, with a minimum of 63.9 °C.

In the temperature range of 200–900 °C, the condensation of silanol groups on the surface of the nanoparticles takes place, accompanied by the elimination of the resulting water molecules—this process being responsible for the formation of silica and its densification. The process takes place slowly, with a low intensity up to 600 °C, then a bit faster—the recorded mass loss being 2.66%. The residual mass was 96.89% (Table 4).

**Figure 8:**
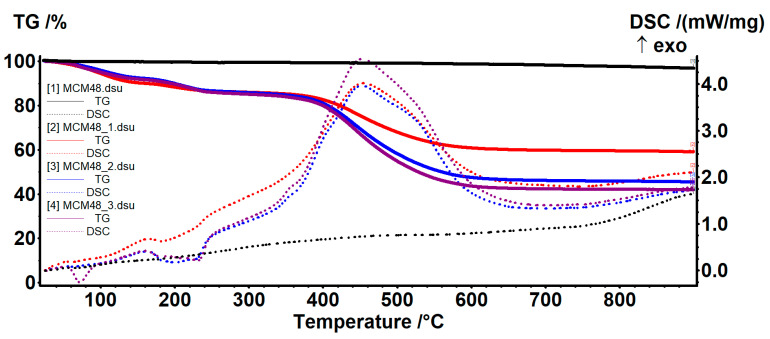
TG-DSC curves for the MCM48 and MCM48-GA samples.

For MCM-48, there are two temperature ranges assigned in the literature for the two processes (water removal and condensation of silanol groups). Thus, at up to 200 °C, the sample loses the water molecules physically absorbed on its surface and in its pores, as well as the water molecules that physically interact with the surface of the nanoparticles—mainly through hydrogen bonds. The mass loss recorded in the range of 20–200 °C was 0.36%. At up to ~100 °C, the sample will mainly lose water unrelated to nanoparticles or which has very weak interactions—0.24%. In the range of 100–200 °C, the water molecules that interact with the surface of the MCM-48 are eliminated, and therefore require a higher energy to be released—0.12%. The release of these two types of water molecules was highlighted by the endothermic effect on the DSC curve, with a minimum of 64.7 °C (Figure 8).

In the temperature range of 200–900 °C, the condensation of silanol groups on the surface of the nanoparticles takes place, accompanied by the elimination of the resulting water molecules—this process being responsible for the formation of silica and its densification. The process takes place slowly, with a low intensity, appearing in the form of a slow mass loss of 2.77% and continuing throughout the interval. The residual mass was 96.86%.

The density of H_2_O molecules and –OH moieties were calculated as indicated in [52]. The amount of H_2_O molecules and –OH moieties per 1 g MCM41/MCM48 was expressed as n_H_2_O_ and n_OH_ and were calculated from the equation:

n_OH_ = 2n_H_2_O_ = 2 (W_T_0__ − W_T_fin__)/(100 × M_H_2_O_)

where W_T_0__ − W_T_fin__ is the weight loss (wt.%) in the temperature range T_0_ − T_fin_; M_H_2_O_—water molecular mass.

The number of water molecules/–OH groups (N_H_2_O_; N_OH_) per 1 nm^2^ were calculated from the equation:

N = n × N_A_ × 10^−18^/S

where n is the amount of water/–OH groups (mmol/g); N_A_—Avogadro number, S—Specific surface area of the sample from BET.

The thermal analysis also provided information about the thermal stability of the GA-loaded samples and permitted an estimation of the % of GA loaded on each sample (Table 5).

The GA-loaded MCM41/MCM48 samples exhibited similar thermal behaviors. The elimination of absorbed water molecules took place between RT–160 °C. The process was accompanied by an endothermic effect, with a minimum at around 60–65 °C. The samples suffered a further degradative process of their organic portion at between 160–300 °C, with increasing mass loss as GA% increased (Table 5). In this interval, two endothermic effects can be noticed on the DSC curve at around 180 °C and over 210 °C, assigned to the decomposition of the GA molecules that present interactions with the MCM supports and the free molecules, respectively. Above 300 °C, the complete oxidation of the organic residues took place, which continued at up to 700 °C. The process was accompanied by a strong, large, and asymmetric exothermic effect, with a maximum at about 450 °C. The residual mass was used to estimate the GA content for each sample.

### 3.7. Release Studies

**Figure 9:**
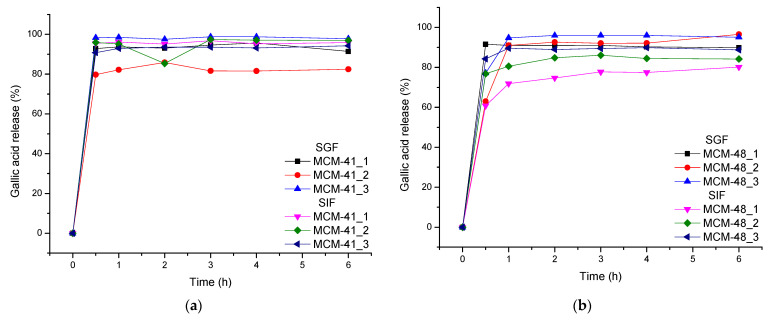
Gallic Acid release from mesoporous support types (**a**) MCM-41 and (**b**) MCM-48 in SGF and SIF.

Figure 9 represents the release profiles of gallic acid from the mesoporous silica supports in two types of biological fluids: simulated gastric fluid (pH = 1.2) and simulated intestinal fluid (pH = 6.8). Paying attention to the release curves, it can be seen that the release was rapid in both biological fluids. It can be seen that according to the simulated fluid used and the nature of the mesoporous support, the release behavior of the drug delivery systems was different. For instance, it is obvious that MCM-41_2 showed the lowest release (~82%) in SGF (after 6 h), while for the MCM-48, the lowest release was obtained in SIF for sample MCM-48_1.

Based on the delivery curves of the two sets of drug delivery systems, significant differences can be observed. For instance, it is important to mention that, depending on the simulated body fluid used, the release behavior of the GA from the two types of mesoporous materials was different. In SGF, the release of the GA from MCM-48 was quite independent of the ratio between the support and the GA (Figure 9b), but significant differences were observed in the case of MCM-41-based systems (Figure 9a). Oppositely, in SIF, the release was independent for MCM-41 and important differences could be observed in the case of MCM-48.

In SGF (pH = 1.2), the recovery of gallic acid from the systems with MCM-41 and MCM-48 could be seen to be over ~80% after one hour and increased at ~95% after 6 h—except with MCM-41_2, where a recovery of up to ~82% was observed.

In the case of SIF (pH = 6.8), it could be seen that the recovery of gallic acid from MCM-41 systems was over ~95% after six hours, and that from systems with MCM-48, the recovery of gallic acid was lower—80–90% after 6 h.

Based on the literature, mesoporous silica materials have been exploited for many medical applications, including cancer treatment. For instance, Gai et al. [53] developed some mesoporous systems loaded with doxorubicin and found a release profile in PBS similar to that obtained by us in SGF for MCM-41 and SIF for MCM-48, respectively. Thus, they reported a rapid release in the first hour, followed by a sustained release (~80% in 5 h). On the other hand, Sanson et al. [54] evaluated the release of the same drug from a biodegradable polymersome based on poly(trimethylene carbonate)-b-poly(L-glutamic acid) and found that the release could be easily extended for a longer period of time, and also that the pH can be used as a proper triggering factor.

Based on the release profiles, the two types of mesoporous materials show different release profiles for GA in SGF or SIF. As the release of gallic acid is complete within 1 h for MCM-41 in SIF and MCM-48 in SGF, but also as the release of the GA from the other two systems is fast, two optimization approaches may be considered. First, the surface of the mesoporous materials could be functionalized with different functional groups or, second, they could be embedded into bioactive polymers for a further tuning of the GA release [55,56,57].

### 3.8. Biological Evaluation

#### 3.8.1. Evaluation of the Antibacterial Effects of Gallic Acid

Testing for the antimicrobial effects of gallic acid was performed by the micro-dilution method in broth using 96-well plates and reading at 620 nm. The chessboard method was performed to establish the synergic effects of GA with amoxicillin (AMX). Lower gallic acid concentrations did not affect the evaluated bacteria (noting only an impact on *E. coli* 25922 and *Shigella* 12022). By increasing the concentration to 500 µg/mL the effects of GA combinations with mesoporous materials were potentiated (Table 6).

#### 3.8.2. Cell Viability

**Figure 10:**
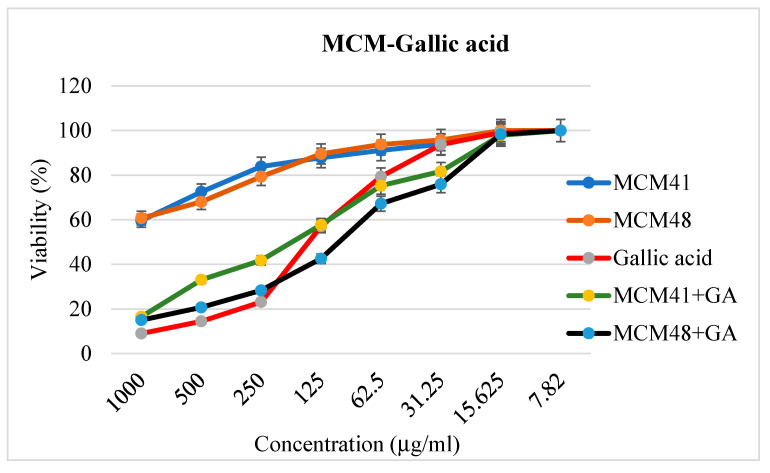
Characterization of prebiotic-induced toxic effects in HT29 cells using the CellTiter-Glo^®^ Luminescent Cell Viability Assay.

The CellTiter-Glo^®^ Luminescent Cell Viability Assay was used to measure ATP as an indicator of cell viability. The 50% inhibitory concentration (IC_50_) was calculated for each gallic acid and their combinations with mesoporous materials based on the luminescence measured on a multi-reader Tristar2S LB942. Thus, the calculated IC_50_ was 125.89 µg/mL for gallic acid, 177.95 µg/mL for MCM41-gallic acid, and 106.82 µg/mL for MCM48-gallic acid (Figure 10). Based on these results, it can be concluded that cell viability is maintained at over 50% for the pure support materials over the whole evaluation range (1000–7.82 µg/mL). It is worth mentioning that both mesoporous substrates had very low toxicity compared to GA-loaded systems. This indicates the possibility of using these materials as a drug delivery support without affecting the cells and the cellular microenvironment. On the other hand, the results demonstrate the efficacy of GA against antitumor cells—its effects as an antitumor and adjuvant of antitumor drugs being well known [29,58,59]

#### 3.8.3. Inflammasome Activation

Inflammasome activation was evaluated using a Caspase-Glo^®^ Inflammasome Assay on HT29 cells. On the one hand, we assessed the effects of gallic acid and its combinations with MCM-41 and MCM-48. On the other hand, we evaluated the impact of bacterial infections in the presence of gallic acid–mesoporous material combinations.

**Figure 11:**
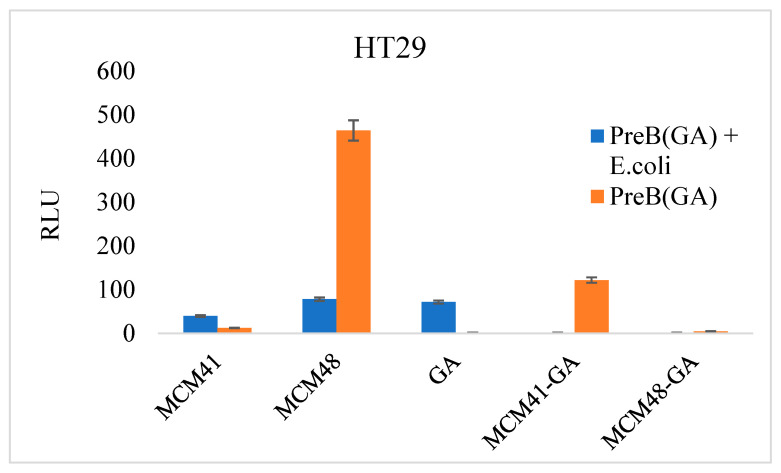
Inflammasome activation in cells infected with prebiotic-treated bacteria (HT29 cells). The signal-dependent activation is shown in red (prebiotic-GA) and blue (E. coli treated with prebiotic). Cell autofluorescence was eliminated by subtracting the RLU of the control cells from the RLU value of each experimental point.

The expression level of inflammatory caspases was greatly increased in the case of mesoporous materials without the addition of prebiotics. This demonstrates that MCM-41 and MCM-48 do not protect against bacterial infection, as the RLU level was high. It should be noted that prebiotics also induced caspase activation in HT29 cells at the tested concentration. Additionally, at the tested concentration, GA probably failed to inhibit the bacterial infection completely, and thus, a slight activation of the caspase of the inflammasome was observed (Figure 11). GA–mesoporous material combinations inhibited the infection and did not activate the inflammasome under the tested conditions.

**Figure 12:**
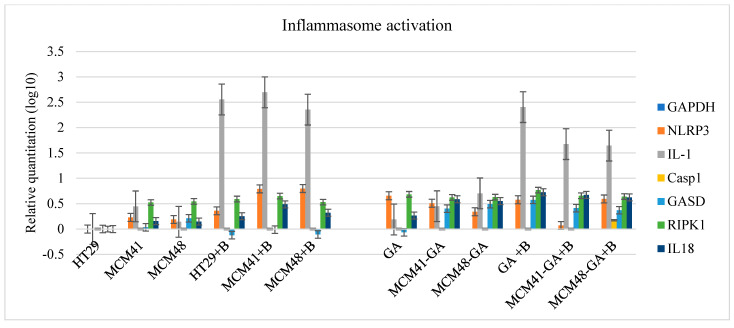
The mRNA expression level of the molecules involved in inflammation.

Next, we wanted to see what happens at the molecular level. There are some subsets of inflammasomes whose assembly is initiated by different stimuli. NLRP3, a member of the Nod-like receptor (NLR) inflammasome family, is activated by a broad range of microbial pathogens and danger-associated molecular patterns, such as crystalline substances [60]. Thus, in our experiments, the levels of NLRP3 mRNA fluctuated without any association with the presence of bacteria or substances. Nevertheless, inflammasomes control the maturation and secretion of proinflammatory cytokines interleukin (IL)-1β and IL-18 to induce pyroptosis, the inflammatory cell death process [61]. At the mRNA level, although the expression level of caspase 1 was very low, there was an increase in the expression level of IL-1b, one of the main actors in inflammation. IL-1b expression was increased in the presence of bacteria (*p* < 0.05). However, in Figure 12 we can observe that in bacterial infection, IL-1b levels were slightly reduced in the presence of gallic acid-loaded MCM compared to its levels when it acted alone, which could be due to the ability of MCM to ameliorate the effects of bacterial infection based on its time-dependent release. IL-18 levels were higher in the presence of bacteria, but also in the presence of GA. As shown in Figure 12, gasdermin (GASD)—another crucial component of inflammasomes—might be associated with the presence of GA.

#### 3.8.4. Apoptosis Activation

**Figure 13:**
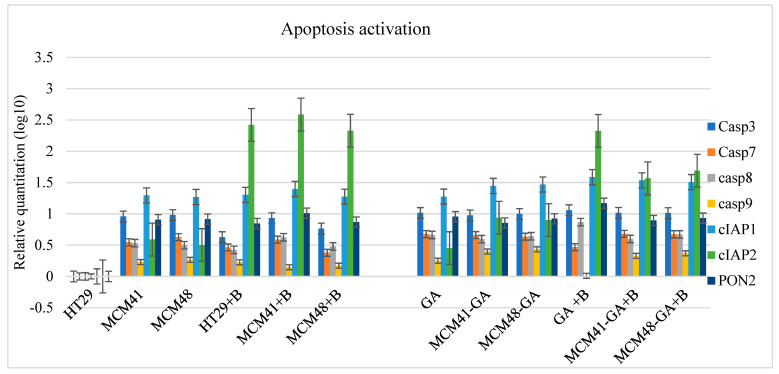
The mRNA expression levels of the molecules involved in apoptosis.

As shown in Figure 13, all apoptotic caspases were activated by drugs and bacterial infection. The levels of Caspase 9 mRNA were low but occurred at all experimental points, sustaining a slight activation of intrinsic apoptosis—Caspase 9 being activated post-cytochrome c release. Additionally, Caspase 9 activates effector caspases and Bid to remodel the mitochondria [62]. Caspase 8 was also activated, showing the activation of the extrinsic apoptotic pathway and effectors Caspase 3 and 7.

Figure 13 shows the increase in the cellular inhibitor of apoptotic cell death 2 (cIAP2), a molecule that inhibits cell death by directly repressing the pro-apoptotic activity of caspases and targeting the pro-apoptotic components of the TNF-pathway for ubiquitinylation degradation [63]. Under normal conditions, an inflammatory response is beneficial in controlling pathogens, but a hyperinflammatory response can lead to pathogenic endotoxic shock [64]. We observed that in the presence of the bacterium, the cIAP2 expression level was increased. In that case, it decreased slightly in the combination of MCM-GA + bacterium, which indicates the GA’s role in reducing the infection. It should be noted that cIAP2 presented the same profile of expression as IL-b1, accounting for the Il-1b secretion through cIAP2’s regulation of caspase-1.

Based on several works, it is imperative to have a fine balance, because aberrant activation can lead to diseases such as autoinflammatory disorders, cardiometabolic diseases, or even cancer [65]. According to Chen G. [66], the inflammasomes play important roles in regulating the gut microbiome.

**Figure 14:**
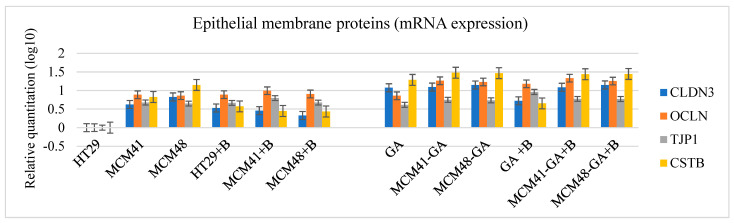
The mRNA expression levels of some cellular membrane molecules.

In addition, we quantified the mRNA expression levels of some membrane proteins: claudin 3 (CLDN3), occludin (OCLN), tight junction protein 1 (TJP1), and si cystatin B (CSTB; Figure 14), and observed an increased association with the extrinsic activation of cells.

## 4. Conclusions

Two types of mesoporous silica materials, MCM-41 and MCM-48, were obtained with a large surface area (1179 and 1482 m^2^/g, respectively). A major finding of this work is related to the modified loading technique developed. First, the mesoporous materials are vacuumed (0.1 barr) for 30 min to remove the adsorbed air and water from the pores, followed by the addition of the GA solution. The solution volume should be similar to the volume of the wet mesoporous support and, in this case, after the pressure is changed to atmospheric pressure, these solutions are absorbed inside the pores, and thus the GA will be mostly disposed inside them. Due to the important differences from the point of view of the surface area, the release profile was different, and thus, the biological responses of the studied bacterial strains were different. Lower gallic acid concentrations only affected *E. coli* 25922 and *Shigella* 12022, and a high concentration of GA-MCM combinations potentiated its antibacterial effects. Mesoporous substrates had very low toxicity compared to GA-loaded systems, indicating the usefulness of using these materials as a drug delivery support without affecting the cells. On the other hand, our results demonstrate that combination with MCM does not decrease the efficacy of GA against antitumor cells. The caspase inflammasome was activated by bacterial infection, and MCM-41 and MCM-48 demonstrated that they do not protect against bacterial infection. The slight reduction in IL-1b in the presence of GA-loaded mesoporous systems was probably due to the gradual release of GA. Taken together, all of these results suggest that gradual release systems potentiate antibacterial activity against some Gram-negative bacteria, but further research is needed to demonstrate the fine-grained mechanisms of bacterial infection.

## Figures and Tables

**Table 1:** Synthesized MCM/gallic acid mesoporous materials.

Sample Code	Materials Type	Mass Ratio MCM-X:Gallic Acid
MCM-41	MCM-41	
MCM-41_1	MCM-41: Gallic acid	1:0.41
MCM-41_2	MCM-41: Gallic acid	1:0.82
MCM-41_3	MCM-41: Gallic acid	1:1.21
MCM-48	MCM-48	
MCM-48_1	MCM-48: Gallic acid	1:0.41
MCM-48_2	MCM-48: Gallic acid	1:0.82
MCM-48_3	MCM-48: Gallic acid	1:1.21

**Table 2:** Working conditions used in the release of gallic acid from mesoporous silica.

Sample Code	Total Mass Used (mg)	Mass of MCM-X (mg)	Mass of GA (mg)	Volume SGF/SIF (mL)
MCM-41_1	50	35.46	14.54	140
MCM-41_2	50	27.47	22.53	140
MCM-41_3	50	22.62	27.38	140
MCM-48_1	50	35.46	14.54	140
MCM-48_2	50	27.47	22.53	140
MCM-48_3	50	22.62	27.38	140

**Table 3:** BET characteristics of mesoporous materials.

Sample Code	S_BET_ (m^2^/g)	V_pors_ (cm^3^/g)
MCM-41	1179.63	0.7634
MCM-41_1	666.16	0.3421
MCM-41_2	536.30	0.2800
MCM-41_3	384.63	0.2039
MCM-48	1482.00	0.7486
MCM-48_1	585.85	0.3641
MCM-48_2	458.98	0.2870
MCM-48_3	348.147	0.1897

**Table 4:** MCM-41/MCM-48 characteristics from the TGA values.

Sample	Mass Loss% RT-200 °C	Mass Loss% 200–900 °C	Endo Effect ( °C)	Residual Mass % (90 °C)	n_H2O_ (mmol/g)	n_OH_ (mmol/g)	N_H2O_ (Groups/nm^2^)	N_OH_ (Groups/nm^2^)
MCM-41	0.57	2.66	63.9	96.89	0.32	2.96	0.16	1.51
MCM-48	0.36	2.77	64.7	96.86	0.20	3.08	0.08	1.25

**Table 5:** Estimation of mesoporous material loading (using residual masses).

Sample	Mass Loss (%) RT-160 °C	Mass Loss (%) 160–300 °C	Mass Loss (%) 300–700 °C	Residual Mass (%)	GA Content (%)
MCM-41_1	8.56	4.93	25.49	60.52	37.54%
MCM-41_2	6.77	6.91	40.12	45.78	52.75%
MCM-41_3	5.75	7.41	48.40	38.12	60.66%
MCM-48_1	10.03	4.06	26.22	59.06	39.03%
MCM-48_2	7.83	6.46	39.73	45.46	53.07%
MCM-48_3	8.40	6.59	42.81	41.79	56.86%

**Table 6:** The CMI was established for AMX with and without the addition of prebiotics.

		*E. coli 25922*	*E. coli ESBLGG3048*	*Pseudomonas aeruginosa 730*	*Pseudomonas aeruginosa 1430*	*Enterococus faecium 1422*	*Salmonella tiphymurium*	*Enterococcus faecium 2853*	*Shigella flexneri 12022*
AMX		<3.9	>1000	>1000	>1000	>1000	>1000	>1000	<2
Gallic acid	150 µg/mL	<7.81	>1000	>1000	>1000	>1000	>1000	>1000	<3.9
	500 µg/mL	<7.81	>1000	>1000	>1000	>1000	>1000	>1000	<2
MCM41-gallic acid	150 µg/mL	<7.81	>1000	>1000	>1000	>1000	>1000	>1000	<2
	500 µg/mL	<1	<1	<1	<1	>1000	<1	<1	<1
MCM48-gallic acid	150 µg/mL	<1.95	>1000	>1000	>1000	>1000	>1000	>1000	<2
	500 µg/mL	<1	<1	<1	<1	>1000	<1	<1	<1

## Data Availability

Available on demand.
